# Supplementary material for: Over 90 endangered fish and invertebrates are caught in industrial fisheries
Source: Nat Commun. 2020 Sep 21;11:4764. doi: 10.1038/s41467-020-18505-6 (PMC7506527; doi:10.1038/s41467-020-18505-6)
Supplement: Supplementary file 1 — Supplementary Information [file 41467_2020_18505_MOESM1_ESM.pdf]

**Title:** *Over ninety endangered fish and invertebrates are caught in industrial fisheries*

Roberson et al.

**Supplementary Information**

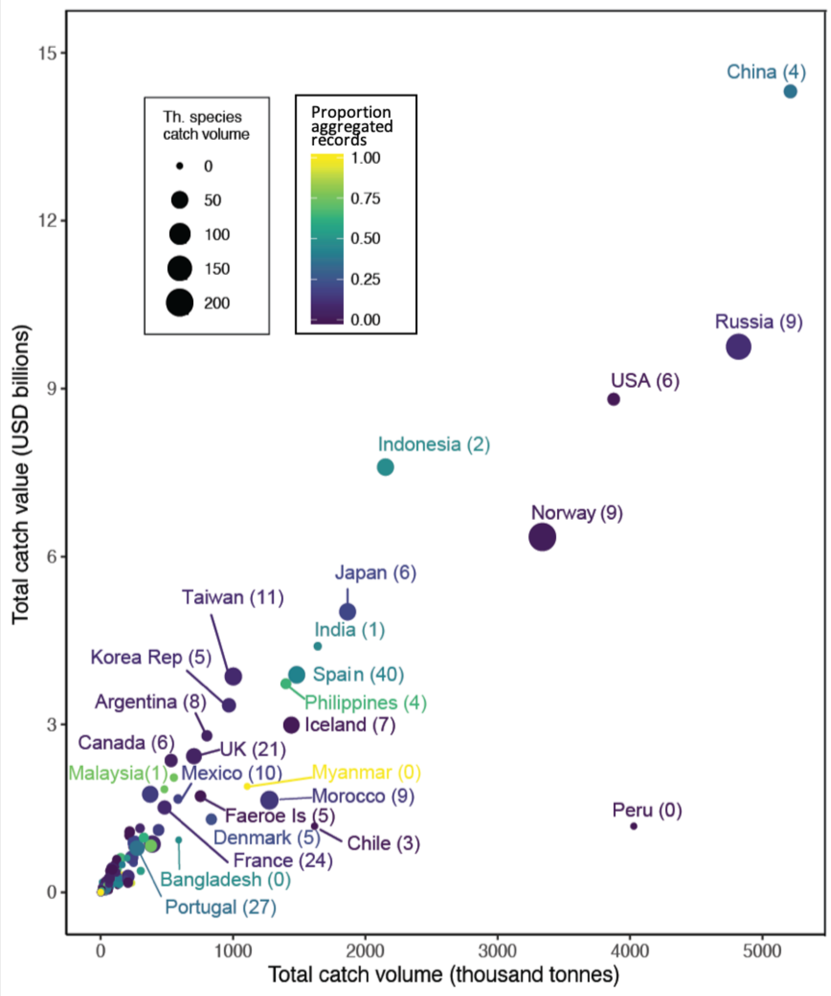


**Supplementary Figure 1:** Catch volume and estimated value for 181 fishing countries in the global catch database described in Watson & Tidd (2018). Bubble size corresponds to volume of threatened species catch. Number of threatened species each country catches is in parentheses. Colour shows the ratio of volume of aggregated records to volume of species-level records (i.e., yellow indicates catch volumes mostly recorded in aggregated and purple indicates catch volumes mostly recorded to the species-level). Volumes and values are 5-year weighted moving averages for 2010

**Supplementary Table 1:** Red List assessment and fishing information for the threatened species appearing in global catch data. Chond = chondrichthyan, Invert = invertebrate. Cat = Category, CR = Critically Endangered, EN = Endangered, VU = Vulnerable). Threats were coded as 1 = Targeted industrial fishing, 2 = Incidental industrial fishing, 3 = Targeted non-industrial fishing, 4 = Incidental non-industrial fishing, 5 = Unspecified fishing, 6 = Other. Price is mean ex-vessel price over the time period (2006 - 2014). Species in bold are listed in the RAM Stock Legacy Database. Species highlighted in grey were last assessed before 2010. *Gadus morhua* was excluded from the final analysis of threatened species

| Species | Taxon group | Red List Assessments | | | | Appendices | | Price (USD/kg) | Countries (num.) | |
|---|---|---|---|---|---|---|---|---|---|---|
| | | Cat. | Year | Pop. trend | Threats | CITES | CMS | | Fishers | Importers |
| *Acipenser gueldenstaedtii* | Teleost | CR | 2009 | Decreasing | 1,2,3,4,6 | II | II | 1.1 | 1 | -- |
| *Acipenser stellatus* | Teleost | CR | 2009 | Decreasing | 1,3,6 | II | II | 1.1 | 1 | -- |
| *Acipenser sturio* | Teleost | CR | 2009 | Decreasing | 2,4,6 | I | I/II | 1.4 | 1 | -- |
| *Alopias superciliosus* | Chond. | VU | 2007 | Decreasing | 1,2,3,4,6 | II | II | 0.4 | 19 | -- |
| *Alopias vulpinus* | Chond. | VU | 2007 | Decreasing | 1,2,3,4,6 | II | II | 0.8 | 20 | -- |
| *Alosa aestivalis* | Teleost | VU | 2011 | Decreasing | 6 | -- | -- | 1.2 | 1 | -- |
| *Alosa immaculata* | Teleost | VU | 2008 | Decreasing | 1,3,6 | -- | -- | 0.9 | 4 | -- |
| *Anguilla anguilla* | Teleost | CR | 2013 | Decreasing | 1,3,5,6 | II | II | 9.5 | 18 | -- |
| *Anguilla rostrata* | Teleost | EN | 2013 | Decreasing | 1,6 | -- | -- | 7.7 | 2 | -- |
| *Apostichopus japonicus* | Invert. | EN | 2010 | Decreasing | 1,3 | -- | -- | 2.1 | 3 | 88 |
| *Atlantoraja cyclophora* | Chond. | VU | 2006 | Decreasing | 2,3 | -- | -- | 2.3 | 1 | -- |
| ***Balistes capriscus*** | **Teleost** | **VU** | **2011** | **Decreasing** | **1,3** | **--** | **--** | **1.7** | **8** | **--** |
| *Bolbometopon muricatum* | Teleost | VU | 2007 | Decreasing | 3,6 | -- | -- | 4.2 | 1 | -- |
| *Carcharhinus albimarginatus* | Chond. | VU | 2015 | Decreasing | 1,2,3,4 | -- | -- | 0.5 | 1 | -- |
| *Carcharhinus dussumieri* | Chond. | EN | 2018 | Decreasing | 2,3,4 | -- | -- | 1.5 | 2 | -- |
| *Carcharhinus falciformis* | Chond. | VU | 2017 | Decreasing | 2,4 | II | II | 0.8 | 31 | -- |
| *Carcharhinus longimanus* | Chond. | VU | 2006 | Decreasing | 1,2,3,4 | II | -- | 0.8 | 31 | -- |
| ***Carcharhinus obscurus*** | **Chond.** | **VU** | **2007** | **Decreasing** | **1,2,3,4,6** | **--** | **II** | **1.5** | **10** | **--** |
| ***Carcharhinus plumbeus*** | **Chond.** | **VU** | **2007** | **Decreasing** | **1,2,3,4,6** | **--** | **--** | **0.9** | **7** | **--** |
| *Carcharias taurus* | Chond. | VU | 2005 | Unknown | 1,2,4,6 | -- | -- | 4.9 | 6 | -- |

**Supplementary Table 1 cont.**

| | | | | | | | | | | |
|---|---|---|---|---|---|---|---|---|---|---|
| *Carcharodon carcharias* | Chond. | VU | 2005 | Unknown | 2,3,4,5,6 | II | I/II | 2.1 | 11 | -- |
| *Centrophorus lusitanicus* | Chond. | VU | 2008 | Unknown | 2,3,4 | -- | -- | 3.3 | 2 | -- |
| ***Centrophorus squamosus*** | **Chond.** | **VU** | **2003** | **Decreasing** | **2,4** | **--** | **--** | **1.5** | **11** | **--** |
| ***Cetorhinus maximus*** | **Chond.** | **VU** | **2005** | **Decreasing** | **2,4,5,6** | **II** | **I/II** | **2.1** | **10** | **--** |
| ***Coryphaenoides rupestris*** | **Teleost** | **CR** | **2012** | **Unknown** | **1** | **--** | **--** | **1.4** | **14** | **--** |
| *Cymatoceps nasutus* | Teleost | VU | 2009 | Decreasing | 1,3 | -- | -- | 4.3 | 1 | -- |
| ***Dalatias licha*** | **Chond.** | **VU** | **2017** | **Decreasing** | **1,2,3,4** | **--** | **--** | **1** | **11** | **--** |
| *Dentex dentex* | Teleost | VU | 2009 | Unknown | 1,3,6 | -- | -- | 17.1 | 12 | -- |
| *Dipturus batis* | Chond. | CR | 2006 | Decreasing | 2,4 | -- | -- | 1.7 | 6 | -- |
| ***Epinephelus itajara*** | **Teleost** | **VU** | **2016** | **Decreasing** | **1,3,4,6** | **--** | **--** | **11.9** | **3** | **--** |
| *Epinephelus marginatus* | Teleost | VU | 2016 | Decreasing | 1,3 | -- | -- | 11.4 | 13 | -- |
| ***Epinephelus morio*** | **Teleost** | **VU** | **2016** | **Decreasing** | **1,3,6** | **--** | **--** | **4.4** | **3** | **--** |
| *Epinephelus striatus* | Teleost | CR | 2016 | Decreasing | 1,3,6 | -- | -- | 8.4 | 2 | 11 |
| ****Gadus morhua*** | **Teleost** | ***VU** | **1996** | **Unknown** | **--** | **--** | **--** | **2.9** | **24** | **179** |
| ***Galeorhinus galeus*** | **Chond.** | **VU** | **2006** | **Decreasing** | **1,2,3,4** | **--** | **--** | **1.6** | **20** | **--** |
| *Gymnura altavela* | Chond. | VU | 2007 | Decreasing | 2,3,4 | -- | -- | 2.3 | 2 | -- |
| ***Hippoglossus hippoglossus*** | **Teleost** | **EN** | **1996** | **Unknown** | **--** | **--** | **--** | **9** | **20** | **153** |
| *Hyporthodus flavolimbatus* | Teleost | VU | 2016 | Decreasing | 1,2,3 | -- | -- | 5.8 | 3 | -- |
| *Hyporthodus niveatus* | Teleost | VU | 2016 | Decreasing | 1,3 | -- | -- | 6.1 | 2 | -- |
| ***Isurus oxyrinchus*** | **Chond.** | **EN** | **2018** | **Decreasing** | **1,2,3,4,5,6** | **II** | **II** | **2.9** | **45** | **--** |
| *Isurus paucus* | Chond. | EN | 2018 | Decreasing | 1,2,3,4 | II | II | 1.1 | 10 | -- |
| *Kajikia albida* | Teleost | VU | 2010 | Decreasing | 1,2,3,4 | -- | -- | 2.9 | 22 | 19 |
| ***Lamna nasus*** | **Chond.** | **VU** | **2006** | **Decreasing** | **1,2,3,4** | **II** | **II** | **3.4** | **33** | **--** |
| *Lethrinus mahsena* | Teleost | EN | 2018 | Decreasing | 1 | -- | -- | 4.4 | 3 | -- |

**Supplementary Table 1 cont.**

| | | | | | | | | | | |
|---|---|---|---|---|---|---|---|---|---|---|
| ***Leucoraja circularis*** | **Chond.** | **EN** | **2014** | **Decreasing** | **2,4** | **--** | **--** | **2.4** | **5** | **--** |
| ***Leucoraja fullonica*** | **Chond.** | **VU** | **2014** | **Decreasing** | **2,4** | **--** | **--** | **2.1** | **5** | **--** |
| *Limulus polyphemus* | Invert. | VU | 2016 | Decreasing | 1,3,6 | -- | -- | 1.3 | 1 | -- |
| ***Lopholatilus chamaeleonticeps*** | **Teleost** | **EN** | **2013** | **Decreasing** | **1,6** | **--** | **--** | **4.9** | **1** | **--** |
| ***Lutjanus campechanus*** | **Teleost** | **VU** | **2015** | **Decreasing** | **1,3** | **--** | **--** | **4.8** | **2** | **--** |
| ***Makaira nigricans*** | **Teleost** | **VU** | **2010** | **Decreasing** | **1,2,3,4** | **--** | **--** | **1.9** | **30** | **51** |
| *Megalops atlanticus* | Teleost | VU | 2018 | Decreasing | 2,3,4,6 | -- | -- | 0.8 | 4 | -- |
| ***Melanogrammus aeglefinus*** | **Teleost** | **VU** | **1996** | **Unknown** | **--** | **--** | **--** | **1.7** | **18** | **181** |
| *Merluccius senegalensis* | Teleost | EN | 2012 | Decreasing | 1,3,6 | -- | -- | 2.2 | 6 | 70 |
| *Mobula mobular* | Chond. | EN | 2014 | Decreasing | 1,2,3,4 | II | I/II | 0.7 | 1 | -- |
| *Mola mola* | Teleost | VU | 2011 | Decreasing | 1,2 | -- | -- | 1.9 | 12 | -- |
| *Mustelus mustelus* | Chond. | VU | 2004 | Decreasing | 2,4 | -- | -- | 2.3 | 16 | -- |
| *Mustelus schmitti* | Chond. | EN | 2006 | Decreasing | 1,2,4 | -- | -- | 2.6 | 2 | -- |
| *Mycteroperca interstitialis* | Teleost | VU | 2016 | Decreasing | 1,3 | -- | -- | 3 | 1 | -- |
| ***Mycteroperca microlepis*** | **Teleost** | **VU** | **2016** | **Decreasing** | **1,3** | **--** | **--** | **8.2** | **2** | **--** |
| *Nebrius ferrugineus* | Chond. | VU | 2003 | Decreasing | 2,4 | -- | -- | 0.6 | 1 | -- |
| *Nemipterus virgatus* | Teleost | VU | 2009 | Decreasing | 1 | -- | -- | 2.1 | 3 | -- |
| *Oxynotus centrina* | Chond. | VU | 2007 | Unknown | 2 | -- | -- | 1.5 | 2 | -- |
| *Palinurus elephas* | Invert. | VU | 2013 | Decreasing | 1 | -- | -- | 15.5 | 9 | -- |
| *Pentanemus quinquarius* | Teleost | VU | 2014 | Decreasing | 2,3,4 | -- | -- | 10.5 | 7 | -- |
| *Plectropomus areolatus* | Teleost | VU | 2016 | Decreasing | 1,3,6 | -- | -- | 3.9 | 1 | -- |
| *Plectropomus pessuliferus* | Teleost | VU | 2016 | Decreasing | 1,3 | -- | -- | 7.5 | 1 | -- |
| ***Pomatomus saltatrix*** | **Teleost** | **VU** | **2014** | **Decreasing** | **1,2,3,4** | **--** | **--** | **3.6** | **23** | **--** |
| *Pseudotolithus senegalensis* | Teleost | EN | 2009 | Decreasing | 1,3,6 | -- | -- | 1.4 | 9 | -- |

**Supplementary Table 1 cont.**

| | | | | | | | | | | |
|---|---|---|---|---|---|---|---|---|---|---|
| *Pseudotolithus senegallus* | Teleost | VU | 2014 | Decreasing | 1,3,6 | -- | -- | 1.1 | 2 | -- |
| *Pseudupeneus prayensis* | Teleost | VU | 2013 | Decreasing | 1,2,3 | -- | -- | 1.9 | 13 | -- |
| ***Raja undulata*** | **Chond.** | **EN** | **2003** | **Decreasing** | **2,4** | **--** | **--** | **2.7** | **4** | **--** |
| *Rhincodon typus* | Chond. | EN | 2016 | Decreasing | 1,2,3,4,6 | II | I/II | 0.6 | 1 | -- |
| ***Rhomboplites aurorubens*** | **Teleost** | **VU** | **2015** | **Decreasing** | **1,3,6** | **--** | **--** | **3.9** | **4** | **--** |
| *Rhynchobatus djiddensis* | Chond. | CR | 2018 | Decreasing | 1,2,3,4,6 | II | -- | 0.8 | 2 | -- |
| *Rostroraja alba* | Chond. | EN | 2006 | Decreasing | 2 | -- | -- | 2.5 | 2 | -- |
| *Sardinella maderensis* | Teleost | VU | 2014 | Unknown | 1,3,6 | -- | -- | 0.6 | 13 | -- |
| *Sciades parkeri* | Teleost | VU | 2011 | Decreasing | 1,3 | -- | -- | 1.3 | 1 | -- |
| ***Sebastolobus alascanus*** | **Teleost** | **EN** | **2000** | **Unknown** | **--** | **--** | **--** | **2.4** | **2** | **--** |
| ***Sphyrna lewini*** | **Chond.** | **EN** | **2007** | **Unknown** | **1,2,3,4,5,6** | **II** | **II** | **0.7** | **18** | **--** |
| *Sphyrna mokarran* | Chond. | EN | 2007 | Decreasing | 1,2,3,4,6 | II | II | 0.5 | 5 | -- |
| *Sphyrna zygaena* | Chond. | VU | 2005 | Decreasing | 1,2,3,4,6 | II | -- | 0.7 | 20 | -- |
| ***Squalus acanthias*** | **Chond.** | **VU** | **2016** | **Decreasing** | **1,2,3,4** | **--** | **II** | **1.6** | **36** | **173** |
| *Squatina argentina* | Chond. | CR | 2017 | Decreasing | 2,4 | -- | -- | 1.3 | 1 | -- |
| ***Squatina squatina*** | **Chond.** | **CR** | **2017** | **Decreasing** | **1,2,3,4,6** | **--** | **I/II** | **1.4** | **7** | **--** |
| *Tautoga onitis* | Teleost | VU | 2008 | Decreasing | 3,6 | -- | -- | 5.8 | 1 | -- |
| ***Thunnus maccoyii*** | **Teleost** | **CR** | **2009** | **Decreasing** | **1** | **--** | **--** | **5.8** | **11** | **57** |
| ***Thunnus obesus*** | **Teleost** | **VU** | **2011** | **Decreasing** | **1,2** | **--** | **--** | **3** | **76** | **193** |
| ***Thunnus orientalis*** | **Teleost** | **VU** | **2014** | **Decreasing** | **1** | **--** | **--** | **7.3** | **23** | **5** |
| ***Thunnus thynnus*** | **Teleost** | **EN** | **2014** | **Decreasing** | **1** | **--** | **--** | **8** | **32** | **127** |
| ***Trachurus mediterraneus*** | **Teleost** | **VU** | **2017** | **Decreasing** | **1,3** | **--** | **--** | **2.4** | **13** | **--** |
| ***Trachurus trachurus*** | **Teleost** | **VU** | **2013** | **Decreasing** | **1,3** | **--** | **--** | **1** | **29** | **133** |
| ***Zearaja chilensis*** | **Chond.** | **VU** | **2007** | **Decreasing** | **1,2,3,4** | **--** | **--** | **2** | **8** | **--** |

**Supplementary Table 2**: IUCN threat codes listed for the 92 threatened species found in the catch data. Codes are categorized into 6 groups ("Code"). Spp = number of species with that threat listed. Threat codes numbered >100 and described as "OLD" are for species last assessed before the updated IUCN threat codes

| **Code** | **Description** | **Spp.** | **IUCN threat code and description** |
|---|---|---|---|
| 1 | Targeted industrial fishing | 65 | 5.4.2 Intentional use: (large scale) [harvest] |
| 1 | Targeted industrial fishing | 65 | 101.4 OLD 3.1.3 Harvesting (hunting/gathering)->Food->Regional/international trade |
| 1 | Targeted industrial fishing | 65 | 101.16 OLD 3.4.3 Harvesting (hunting/gathering)->Materials->Regional/international trade |
| 1 | Targeted industrial fishing | 65 | 5.3.2 Intentional use: (large scale) [harvest] |
| 2 | Incidental industrial fishing | 50 | 5.4.4 Unintentional effects: (large scale) [harvest] |
| 3 | Targeted non-industrial fishing | 61 | 5.4.1 Intentional use: (subsistence/small scale) [harvest] |
| 3 | Targeted non-industrial fishing | 61 | 101.2 OLD 3.1.1 Harvesting (hunting/gathering)->Food->Subsistence use/local trade |
| 3 | Targeted non-industrial fishing | 61 | 101.3 OLD 3.1.2 Harvesting (hunting/gathering)->Food->Sub-national/national trade |
| 4 | Incidental non-industrial fishing | 44 | 5.4.3 Unintentional effects: (subsistence/small scale) [harvest] |
| 5 | Unspecified fishing | 5 | 101.17 OLD 3.5 Harvesting (hunting/gathering)->Cultural/scientific/leisure activities |
| 5 | Unspecified fishing | 5 | 101.1 OLD 3.1 Harvesting (hunting/gathering)->Food |
| 5 | Unspecified fishing | 5 | 5.1.1 Intentional use (species is the target) |
| 6 | Other | 36 | 5.4.5 Persecution/control |
| 6 | Other | 36 | 9.3.4 Type Unknown/Unrecorded |
| 6 | Other | 36 | 6.1 Recreational activities |
| 6 | Other | 36 | 9.1.1 Sewage |
| 6 | Other | 36 | 9.1.3 Type Unknown/Unrecorded |
| 6 | Other | 36 | 8.1.2 Named species |
| 6 | Other | 36 | 101.35 OLD 9.5 Intrinsic factors->Low densities |
| 6 | Other | 36 | 101.32 OLD 9.2 Intrinsic factors->Poor recruitment/reproduction/regeneration |
| 6 | Other | 36 | 3.1 Oil & gas drilling |
| 6 | Other | 36 | 4.3 Shipping lanes |
| 6 | Other | 36 | 7.2.10 Large dams |
| 6 | Other | 36 | 8.2 Problematic native species/diseases |
| 6 | Other | 36 | 7.2.9 Small dams |
| 6 | Other | 36 | 9.2.3 Type Unknown/Unrecorded |
| 6 | Other | 36 | 100.18 OLD 4.1.1 Accidental mortality->Bycatch->Fisheries related |
| 6 | Other | 36 | 101.37 OLD 9.7 Intrinsic factors->Slow growth rates |

**Supplementary Table 2 cont.**

| | | | |
|---|---|---|---|
| 6 | Other | 36 | 101.13 OLD 3.4 Harvesting (hunting/gathering)->Materials |
| 6 | Other | 36 | 1.1 Housing & urban areas |
| 6 | Other | 36 | 7.2.11 Dams (size unknown) |
| 6 | Other | 36 | 8.1.1 Unspecified species |
| 6 | Other | 36 | 12.1 Other threat |
| 6 | Other | 36 | 3.2 Mining & quarrying |
| 6 | Other | 36 | 9.2.1 Oil spills |
| 6 | Other | 36 | 11.1 Habitat shifting & alteration |
| 6 | Other | 36 | 11.3 Temperature extremes |
| 6 | Other | 36 | 9.1.2 Run-off |
| 6 | Other | 36 | 6.3 Work & other activities |
| 6 | Other | 36 | 5.4.6 Motivation Unknown/Unrecorded |
| 6 | Other | 36 | 101.40 OLD 9.10 Intrinsic factors->Other |
| 6 | Other | 36 | 101.39 OLD 9.9 Intrinsic factors->Restricted range |
| 6 | Other | 36 | 9.4 Garbage & solid waste |
| 6 | Other | 36 | 9.6.3 Noise pollution |
| 6 | Other | 36 | 7.3 Other ecosystem modifications |
| 6 | Other | 36 | 1.3 Tourism & recreation areas |
| 6 | Other | 36 | 1.2 Commercial & industrial areas |
| 6 | Other | 36 | 2.4.3 Scale Unknown/Unrecorded |
| 6 | Other | 36 | 11.5 Other impacts |
| 6 | Other | 36 | 9.3.3 Herbicides and pesticides |
| 6 | Other | 36 | 9.2.2 Seepage from mining |
| 6 | Other | 36 | 11.4 Storms & flooding |
| 6 | Other | 36 | 2.2.2 Agro-industry plantations |
| 6 | Other | 36 | 2.3.3 Agro-industry grazing, ranching or farming |
| 6 | Other | 36 | 2.1.3 Agro-industry farming |
| 6 | Other | 36 | 9.6.2 Thermal pollution |
| 6 | Other | 36 | 3.3 Renewable energy |
| 6 | Other | 36 | 9.5.1 Acid rain |
| 6 | Other | 36 | 100.29 OLD 4.2.3 Accidental mortality->Collision->Other |
| 6 | Other | 36 | 100.55 OLD 12 Unknown |
| 6 | Other | 36 | 4.1 Roads & railroads |
| 6 | Other | 36 | 7.2.1 Abstraction of surface water (domestic use) |

**Supplementary Table 2 cont.**

| | | | |
|---|---|---|---|
| 6 | Other | 36 | 7.2.5 Abstraction of ground water (domestic use) |
| 6 | Other | 36 | 7.2.6 Abstraction of ground water (commercial use) |
| 6 | Other | 36 | 7.2.7 Abstraction of ground water (agricultural use) |
| 6 | Other | 36 | 11.2 Droughts |
| 6 | Other | 36 | 7.2.2 Abstraction of surface water (commercial use) |
| 6 | Other | 36 | 7.2.3 Abstraction of surface water (agricultural use) |
| 6 | Other | 36 | 9.3.2 Soil erosion, sedimentation |
| 6 | Other | 36 | 9.3.1 Nutrient loads |

**Supplementary Table 3**: Volume and value of country catch. Catch ranks and proportions are based off the 8-year weighted moving average for 2014. The 50 countries catching the largest volumes of threatened (Th.) species between 2006 - 2014 are shown. Ranks are for all 163 fishing countries. Aggregated = not species-level commodity record.

| Fishing country | Th. species volume | | Th. species value | | Commodities | | Aggregated records (%) |
|---|---|---|---|---|---|---|---|
| | Rank | % of total | Rank | % of total | Th. | All | |
| Norway | 1 | 5.9 | 1 | 8.6 | 8 | 37 | 6.1 |
| Russia | 2 | 2.9 | 3 | 3.9 | 12 | 225 | 11.2 |
| Netherlands | 3 | 18.4 | 11 | 7.9 | 11 | 157 | 6.6 |
| Morocco | 4 | 5.2 | 18 | 3.6 | 12 | 86 | 15.5 |
| Ireland | 5 | 25.9 | 5 | 23.5 | 12 | 162 | 12.3 |
| Iceland | 6 | 4.5 | 4 | 6.0 | 8 | 68 | 20.9 |
| Belize | 7 | 14.0 | 8 | 34.9 | 5 | 70 | 67.0 |
| Mauritania | 8 | 26.2 | 31 | 13.1 | 5 | 75 | 50.6 |
| UK | 9 | 5.9 | 9 | 4.8 | 27 | 275 | 9.1 |
| Unknown | 10 | 4.8 | 10 | 4.8 | 29 | 115 | 5.3 |
| Spain | 11 | 4.3 | 7 | 5.4 | 43 | 410 | 33.3 |
| Turkey | 12 | 5.5 | 16 | 9.0 | 8 | 85 | 3.7 |
| Japan | 13 | 0.8 | 2 | 2.1 | 21 | 267 | 42.7 |
| Philippines | 14 | 1.7 | 15 | 3.1 | 3 | 100 | 53.6 |
| Portugal | 15 | 13.0 | 12 | 17.2 | 39 | 308 | 38.1 |
| France | 16 | 4.7 | 14 | 4.0 | 32 | 333 | 18.8 |
| USA | 17 | 0.4 | 6 | 1.9 | 33 | 271 | 7.4 |
| Canada | 18 | 1.6 | 13 | 1.8 | 11 | 99 | 11.9 |
| Tunisia | 19 | 17.6 | 34 | 7.9 | 5 | 61 | 44.2 |
| Senegal | 20 | 6.4 | 27 | 3.5 | 16 | 113 | 41.0 |
| Taiwan | 21 | 2.7 | 19 | 4.1 | 15 | 123 | 11.2 |
| Ukraine | 22 | 6.2 | 24 | 15.0 | 19 | 227 | 52.6 |
| India | 23 | 0.5 | 39 | 0.3 | 13 | 95 | 69.9 |
| Denmark | 24 | 1.3 | 21 | 1.8 | 8 | 97 | 33.1 |
| China | 25 | 0.1 | 23 | 0.1 | 13 | 195 | 51.1 |
| Latvia | 26 | 5.9 | 30 | 12.0 | 5 | 63 | 42.3 |
| South Korea | 27 | 0.7 | 20 | 1.1 | 24 | 339 | 25.1 |
| Mexico | 28 | 0.7 | 22 | 3.6 | 8 | 53 | 2.9 |
| Ghana | 29 | 10.0 | 32 | 13.3 | 7 | 83 | 11.7 |
| Faeroe Islands | 30 | 1.5 | 25 | 2.0 | 6 | 59 | 44.0 |
| Lithuania | 31 | 5.4 | 35 | 7.5 | 5 | 86 | 34.6 |
| Indonesia | 32 | 0.2 | 29 | 0.2 | 2 | 68 | 57.8 |
| Papua New Guinea | 33 | 3.0 | 38 | 4.5 | 3 | 17 | 0.2 |
| Nigeria | 34 | 4.4 | 41 | 1.8 | 1 | 27 | 73.4 |

**Supplementary Table 3 cont.**

| | | | | | | | |
|---|---|---|---|---|---|---|---|
| Brazil | 35 | 1.5 | 26 | 2.7 | 31 | 225 | 21.4 |
| Ecuador | 36 | 1.7 | 33 | 4.5 | 8 | 46 | 13.9 |
| New Zealand | 37 | 2.4 | 37 | 2.0 | 15 | 191 | 27.1 |
| Marshall Islands | 38 | 6.3 | 42 | 10.0 | 3 | 13 | 0.1 |
| Australia | 39 | 2.6 | 28 | 2.6 | 15 | 246 | 69.5 |
| Kiribati | 40 | 4.5 | 48 | 7.1 | 2 | 11 | 0.0 |
| Sri Lanka | 41 | 2.8 | 56 | 1.2 | 7 | 52 | 81.1 |
| Italy | 42 | 1.3 | 17 | 4.3 | 11 | 137 | 28.7 |
| Micronesia | 43 | 6.9 | 43 | 10.7 | 3 | 12 | 0.5 |
| Germany | 44 | 0.9 | 51 | 1.1 | 8 | 110 | 18.3 |
| Greenland | 45 | 0.8 | 45 | 0.7 | 3 | 31 | 14.2 |
| Costa Rica | 46 | 11.5 | 61 | 6.1 | 4 | 25 | 58.7 |
| Congo Republic | 47 | 17.2 | 46 | 21.6 | 2 | 38 | 34.6 |
| Seychelles | 48 | 8.9 | 60 | 11.5 | 1 | 14 | 3.5 |
| Libya | 49 | 34.9 | 40 | 48.3 | 6 | 34 | 35.9 |
| Namibia | 50 | 0.2 | 50 | 0.6 | 7 | 62 | 32.8 |

**Supplementary Table 4**: Volume and value of country imports. Import ranks and proportions are based off the 8-year weighted moving average for 2014. The 50 countries catching the largest volumes of threatened (Th.) species between 2006 - 2014 are shown. Ranks are for all 204 importing countries. Aggregated = not species-level commodity record.

| Importing country | Th. species volume | | Th. species value | | Commodities | | Aggregated records (%) |
|---|---|---|---|---|---|---|---|
| | Rank | % of total | Rank | % of total | Th. | All | |
| UK | 1 | 8.7 | 2 | 6.5 | 10 | 338 | 28.1 |
| Germany | 2 | 5.3 | 4 | 4.6 | 9 | 327 | 25.2 |
| Nigeria | 3 | 4.7 | 6 | 5.1 | 11 | 308 | 45.8 |
| Belgium | 4 | 9.8 | 9 | 5.5 | 8 | 292 | 33.2 |
| Spain | 5 | 2.2 | 1 | 3.3 | 11 | 368 | 49.4 |
| Denmark | 6 | 2.2 | 8 | 2.5 | 11 | 333 | 35.1 |
| USA | 7 | 1.5 | 3 | 2.1 | 12 | 347 | 50.3 |
| Netherlands | 8 | 1.9 | 13 | 1.7 | 10 | 341 | 35.0 |
| China | 9 | 1.1 | 7 | 1.5 | 11 | 365 | 51.2 |
| New Zealand | 10 | 13.8 | 11 | 10.7 | 8 | 256 | 61.9 |
| Thailand | 11 | 1.3 | 5 | 2.6 | 12 | 359 | 45.0 |
| Sweden | 12 | 1.7 | 15 | 2.0 | 10 | 347 | 33.6 |
| France | 13 | 1.6 | 12 | 1.9 | 12 | 351 | 39.5 |
| Italy | 14 | 2.0 | 10 | 2.5 | 10 | 353 | 41.2 |
| Canada | 15 | 2.6 | 17 | 2.9 | 7 | 271 | 29.1 |
| Mauritius | 16 | 4.8 | 14 | 7.4 | 10 | 297 | 37.8 |
| Portugal | 17 | 2.3 | 16 | 3.4 | 11 | 325 | 35.5 |
| Taiwan | 18 | 1.7 | 18 | 1.9 | 9 | 337 | 51.3 |
| Turkey | 19 | 7.2 | 24 | 6.9 | 9 | 264 | 22.8 |
| Ukraine | 20 | 2.1 | 21 | 3.3 | 7 | 228 | 19.8 |
| Hong Kong | 21 | 1.3 | 19 | 2.2 | 8 | 253 | 56.3 |
| Cote d'Ivoire | 22 | 1.7 | 22 | 3.7 | 7 | 262 | 30.3 |
| Angola | 23 | 5.1 | 31 | 5.5 | 10 | 236 | 49.2 |
| Japan | 24 | 0.3 | 20 | 0.5 | 9 | 247 | 74.9 |
| Belarus | 25 | 2.9 | 32 | 3.9 | 6 | 194 | 17.8 |
| Poland | 26 | 0.9 | 35 | 1.1 | 7 | 263 | 42.2 |
| Peru | 27 | 3.1 | 33 | 4.6 | 9 | 255 | 39.1 |
| Namibia | 28 | 1.1 | 23 | 1.6 | 11 | 300 | 47.6 |
| Norway | 29 | 0.5 | 37 | 0.9 | 9 | 293 | 52.6 |
| Ecuador | 30 | 0.8 | 26 | 1.8 | 10 | 252 | 24.8 |
| South Africa | 31 | 2.0 | 34 | 2.4 | 10 | 310 | 68.6 |
| Australia | 32 | 1.7 | 30 | 2.2 | 8 | 169 | 47.2 |
| Fiji | 33 | 2.7 | 28 | 4.5 | 7 | 181 | 24.4 |
| Greece | 34 | 2.3 | 27 | 3.7 | 11 | 304 | 40.3 |

**Supplementary Table 4 cont.**

| | | | | | | | |
|---|---|---|---|---|---|---|---|
| Cameroon | 35 | 2.0 | 39 | 3.1 | 8 | 222 | 35.1 |
| Korea Rep | 36 | 0.3 | 38 | 0.5 | 9 | 291 | 72.4 |
| Falkland Is | 37 | 3.7 | 29 | 6.0 | 10 | 171 | 14.0 |
| Ghana | 38 | 1.0 | 40 | 1.8 | 9 | 269 | 35.4 |
| UAE | 39 | 1.3 | 36 | 2.1 | 3 | 134 | 59.0 |
| Egypt | 40 | 0.7 | 25 | 2.4 | 9 | 259 | 47.2 |
| Chile | 41 | 1.1 | 42 | 1.5 | 7 | 209 | 40.0 |
| Gabon | 42 | 21.6 | 53 | 21.0 | 7 | 156 | 29.6 |
| Russia | 43 | 0.2 | 44 | 0.4 | 8 | 244 | 57.7 |
| Seychelles | 44 | 2.7 | 41 | 5.2 | 7 | 216 | 64.4 |
| Mexico | 45 | 1.1 | 43 | 1.7 | 10 | 260 | 52.8 |
| Iceland | 46 | 1.8 | 50 | 1.6 | 8 | 277 | 35.7 |
| Brazil | 47 | 0.8 | 54 | 1.2 | 8 | 266 | 37.7 |
| Latvia | 48 | 1.2 | 51 | 1.5 | 5 | 197 | 16.4 |
| Benin | 49 | 2.8 | 48 | 5.1 | 8 | 187 | 49.4 |
| Switzerland | 50 | 2.2 | 47 | 3.0 | 7 | 251 | 39.7 |

**Supplementary Table 5:** Best model of countries' threatened species catch volumes (two-way ANOVA). All catch volumes are 2014 weighted moving averages (8-year window). GDP is 2014 per capita GDP (USD). CI = confidence interval, LL = lower limit, UL = upper limit, Sig = Significance

| Predictor variable | Estimate | Std. Error | *t* | 95% CI | | Pr(>*t*) | Sig. |
|---|---|---|---|---|---|---|---|
| | | | | LL | UL | | |
| (Intercept) | 904 | 1.72E+03 | 0.526 | -2494 | 4302 | 0.5998 | |
| Total catch volume | 0.0100 | 0.0021 | 4.656 | 0.000574 | 0.0142 | 4.40E-07 | *** |
| Aggregated records volume | -0.0151 | 0.0047 | -3.232 | -0.0244 | -0.00587 | 0.00153 | ** |
| GDP | 0.2355 | 0.0737 | 3.197 | 0.0898 | 0.3811 | 0.00172 | ** |

Residual standard error: 15330 on 139 degrees of freedom

Adjusted R-squared: 0.2117

F-statistic: 13.71, p = 6.919e-08

**Supplementary Table 6:** Best model of countries' threatened species import volumes (two-way ANOVA). All import volumes are 2015 weighted moving averages (9-year window). GDP is 2014 per capita GDP (USD). CI = confidence interval, LL = lower limit, UL = upper limit, Sig = Significance

| Predictor variable | Estimate | Std. Error | *t* | 95% CI | | Pr(>*t*) | Sig. |
|---|---|---|---|---|---|---|---|
| | | | | LL | UL | | |
| (Intercept) | 42.8409 | 2.40E+02 | 0.178 | -430.6 | 516.3 | 0.859 | |
| Total imports volume | 0.0522 | 0.0035 | 14.921 | 0.0452 | 0.0590 | <2e-16 | *** |
| Aggregated records volume | -0.0708 | 0.0069 | -10.241 | -0.0843 | -0.0571 | <2e-16 | *** |

Residual standard error: 3175 on 206 degrees of freedom

Adjusted R-squared: 0.6644

F-statistic: 206.9, p < 2.2e-16

**Supplementary Table 7:** Names and Red List categories of 61 threatened species found in the global catch database described in Watson & Tidd (2018). CR = Critically Endangered, EN = Endangered, VU = Vulnerable

| Species | Common name | Taxon group | Red List Category |
|---|---|---|---|
| *Alopias superciliosus* | Bigeye thresher | Chondrichthyan | VU |
| *Alopias vulpinus* | Thintail thresher | Chondrichthyan | VU |
| *Alosa immaculata* | Pontic shad | Teleost | VU |
| *Apostichopus japonicus* | Japanese sea cucumber | Invertebrate | EN |
| *Argyrosomus hololepidotus* | Southern meagre | Teleost | EN |
| *Balistes capriscus* | Grey triggerfish | Teleost | VU |
| *Carcharhinus falciformis* | Silky shark | Chondrichthyan | VU |
| *Carcharhinus longimanus* | Oceanic whitetip shark | Chondrichthyan | VU |
| *Carcharhinus plumbeus* | Sandbar shark | Chondrichthyan | VU |
| *Carcharias taurus* | Sand tiger shark | Chondrichthyan | VU |
| *Carcharodon carcharias* | Great white shark | Chondrichthyan | VU |
| *Centrophorus lusitanicus* | Lowfin gulper shark | Chondrichthyan | VU |
| *Centrophorus squamosus* | Leafscale gulper shark | Chondrichthyan | VU |
| *Cetorhinus maximus* | Basking shark | Chondrichthyan | VU |
| *Dalatias licha* | Kitefin shark | Chondrichthyan | VU |
| *Dentex dentex* | Common dentex | Teleost | VU |
| *Dipturus batis* | Blue skate | Chondrichthyan | CR |
| *Epinephelus marginatus* | Dusky grouper | Teleost | VU |
| *Epinephelus striatus* | Nassau grouper | Teleost | CR |
| *Gadus morhua* | Atlantic cod | Teleost | VU |
| *Galeorhinus galeus* | Tope shark | Chondrichthyan | VU |
| *Gymnura altavela* | Spiny butterfly ray | Chondrichthyan | VU |
| *Hippoglossus hippoglossus* | Atlantic halibut | Teleost | EN |
| *Isurus oxyrinchus* | Shortfin mako | Chondrichthyan | EN |
| *Isurus paucus* | Longfin mako | Chondrichthyan | EN |
| *Kajikia albida/Tetrapturus albidus* | Atlantic white marlin | Teleost | VU |
| *Lamna nasus* | Porbeagle | Chondrichthyan | VU |
| *Lethrinus mahsena* | Sky emperor | Teleost | EN |
| *Leucoraja circularis* | Sandy ray | Chondrichthyan | EN |
| *Leucoraja fullonica* | Shagreen ray | Chondrichthyan | VU |
| *Lutjanus campechanus* | Northern red snapper | Teleost | VU |
| *Makaira nigricans* | Atlantic blue marlin | Teleost | VU |
| *Megalops atlanticus* | Tarpon | Teleost | VU |
| *Melanogrammus aeglefinus* | Haddock | Teleost | VU |
| *Merluccius senegalensis* | Senegalese hake | Teleost | EN |

**Supplementary Table 4 cont.**

| | | | |
|---|---|---|---|
| *Mobula mobular* | Devil fish | Chondrichthyan | EN |
| *Mola mola* | Ocean sunfish | Teleost | VU |
| *Mustelus mustelus* | Smooth-hound | Chondrichthyan | VU |
| *Mustelus schmitti* | Narrownose smoothhound | Chondrichthyan | EN |
| *Nemipterus virgatus* | Golden threadfin bream | Teleost | VU |
| *Oxynotus centrina* | Angular roughshark | Chondrichthyan | VU |
| *Palinurus elephas* | Common spiny lobster | Invertebrate | VU |
| *Pentanemus quinquarius* | Royal threadfin | Teleost | VU |
| *Pomatomus saltatrix* | Bluefish | Teleost | VU |
| *Pseudotolithus senegalensis* | Cassava croaker | Teleost | EN |
| *Pseudotolithus senegallus* | Law croaker | Teleost | VU |
| *Pseudupeneus prayensis* | West African goatfish | Teleost | VU |
| *Raja undulata* | Undulate ray | Chondrichthyan | EN |
| *Rhomboplites aurorubens* | Vermilion snapper | Teleost | VU |
| *Sardinella maderensis* | Madeiran sardinella | Teleost | VU |
| *Sebastolobus alascanus* | Shortspine thornyhead | Teleost | EN |
| *Sphyrna lewini* | Scalloped hammerhead | Chondrichthyan | EN |
| *Sphyrna zygaena* | Smooth hammerhead | Chondrichthyan | VU |
| *Squalus acanthias* | Piked dogfish | Chondrichthyan | VU |
| *Squatina argentina* | Argentine angelshark | Chondrichthyan | CR |
| *Squatina squatina* | Angelshark | Chondrichthyan | CR |
| *Thunnus maccoyii* | Southern bluefin tuna | Teleost | CR |
| *Thunnus obesus* | Bigeye tuna | Teleost | VU |
| *Thunnus orientalis* | Pacific bluefin tuna | Teleost | VU |
| *Thunnus thynnus* | Atlantic bluefin tuna | Teleost | VU |
| *Trachurus trachurus* | Atlantic horse mackerel | Teleost | VU |
