# Supplementary material for: Over 90 endangered fish and invertebrates are caught in industrial fisheries
Source: Nat Commun. 2020 Sep 21;11:4764. doi: 10.1038/s41467-020-18505-6 (PMC7506527; doi:10.1038/s41467-020-18505-6)
Supplement: Supplementary file 3 — Reporting Summary [file 41467_2020_18505_MOESM3_ESM.pdf]

nature research

Corresponding author(s): Leslie A Roberson

Last updated by author(s): 7/29/2020

Reporting Summary

Nature Research wishes to improve the reproducibility of the work that we publish. This form provides structure for consistency and transparency in reporting. For further information on Nature Research policies, seeAuthors & Referees and theEditorial Policy Checklist .

Statistics

For all statistical analyses, confirm that the following items are present in the figure legend, table legend, main text, or Methods section.

n/a	Confirmed	
☒	☐	The exact sample size (*n*) for each experimental group/condition, given as a discrete number and unit of measurement
☒	☐	A statement on whether measurements were taken from distinct samples or whether the same sample was measured repeatedly
☐	☒	The statistical test(s) used AND whether they are one- or two-sided<br>*Only common tests should be described solely by name; describe more complex techniques in the Methods section.*
☐	☒	A description of all covariates tested
☐	☒	A description of any assumptions or corrections, such as tests of normality and adjustment for multiple comparisons
☐	☒	A full description of the statistical parameters including central tendency (e.g. means) or other basic estimates (e.g. regression coefficient) AND variation (e.g. standard deviation) or associated estimates of uncertainty (e.g. confidence intervals)
☐	☒	For null hypothesis testing, the test statistic (e.g. *F*, *t*, *r*) with confidence intervals, effect sizes, degrees of freedom and *P* value noted<br>*Give P values as exact values whenever suitable.*
☒	☐	For Bayesian analysis, information on the choice of priors and Markov chain Monte Carlo settings
☒	☐	For hierarchical and complex designs, identification of the appropriate level for tests and full reporting of outcomes
☒	☐	Estimates of effect sizes (e.g. Cohen's *d*, Pearson's *r*), indicating how they were calculated

Our web collection on statistics for biologists contains articles on many of the points above.

Software and code

Policy information about availability of computer code

Data collection	No data were collected for this study
Data analysis	All analyses were conducted in the free software R (version 3.6.0). The code and data used to produce the figures and tables are provided as .csv and R Markdown files on a public GitHub repository (https://github.com/lroberson/thr_seafood_pub)

For manuscripts utilizing custom algorithms or software that are central to the research but not yet described in published literature, software must be made available to editors/reviewers. We strongly encourage code deposition in a community repository (e.g. GitHub). See the Nature Research guidelines for submitting code & software for further information.

Data

Policy information about availability of data

All manuscripts must include a data availability statement. This statement should provide the following information, where applicable:

- Accession codes, unique identifiers, or web links for publicly available datasets
- A list of figures that have associated raw data
- A description of any restrictions on data availability

Five databases were used in this study: Two publicly available databasees: 1) IUCN Redlist of Threatened Species (https://www.iucnredlist.org; link provided in the manuscript text) and 2) the RAM stock legacy database (https://www.ramlegacy.org/database/; link provided in manuscript text). We also used three private databases that have been published previously and are available upon reasonable request (1) global catch database described in Watson, R. A., Green, B. S., Tracey, S. R., Farmery, A. & Pitcher, T. J. Provenance of global seafood. Fish Fish. 17, 585–595 (2016), 2) global seafood trade database described in Watson, R. A., Green, B. S., Tracey, S. R., Farmery, A. & Pitcher, T. J. Provenance of global seafood. Fish Fish. 17, 585–595 (2016), and 3) the Sea Around Us global catch data, www.seaaroundus.org). We also used information on ex-vessel prices, described in Tai, T. C., Cashion, T., Lam, V. W. Y., Swartz, W. & Sumaila, U. R. Ex-vessel fish price database: Disaggregating prices for low-priced species from reduction fisheries. Front. Mar. Sci. 4, 1–10 (2017). This information is not provided as a separate database; it must be derived from the Sea Around Us catch database. Additionally, we requested the associated threat codes information from the IUCN Red List (https://www.iucnredlist.org). This information can be viewed on the database but cannot be downloaded as part of the freely available data.

All of the figures and tables in the text and supplementary information have associated raw data, which we will make available as csv files on the IMAS data repository (https://data.imas.utas.edu.au/static/landing.html) and on a public GitHub repository.

Field-specific reporting

Please select the one below that is the best fit for your research. If you are not sure, read the appropriate sections before making your selection.

☐ Life sciences ☐ Behavioural & social sciences ☒ Ecological, evolutionary & environmental sciences

For a reference copy of the document with all sections, see nature.com/documents/nr-reporting-summary-flat.pdf

Ecological, evolutionary & environmental sciences study design

All studies must disclose on these points even when the disclosure is negative.

Study description	This study used five existing databases (two public and three private/available upon request) to analyze patterns in catch and trade of threatened fish and invertebrate species in industrial-scale fisheries globally.
Research sample	We included all data from 2006 - 2014 in the catch and 2006-2015 in the trade databases. The ex-vessel price data is derived from the Sea Around Us catch database, which we explain in the text. We then selected various subsets to analyze trends in types of fishing (reported catch, industrial) fishing sectors), taxonomy (e.g. fish and invertebrates), recording group (e.g. species-level record) and threat status (we focused on species listed as Vulnerable, Endangered, or Critically Endangered on the IUCN Red List of Threatened Species). We included all years of IUCN Red List assessments, including before 2006, but include the assessment date in the Supplementary Info.
Sampling strategy	Sampling strategy is not inherently relevant to this study as no data were collected. However, we decided to select 9 and 10-year subsets from the existing databases based off previous studies of large-scale trends in global fishing (e.g., Gephart, J. A. & Pace, M. L. Structure and evolution of the global seafood trade network. Environ. Res. Lett. 10, (2015), Schiller, L., Bailey, M., Jacquet, J. & Sala, E. High seas fisheries play a negligible role in addressing global food security. Sci. Adv. 4, (2018)), and from the metadata for the databases themselves.
Data collection	Data collection procedure is not relevant to this study as no data were collected. The global catch, trade, and stock assessment databases used in this study were derived from global fisheries data provided by FAO (http://www.fao.org/fishery/statistics/collections/en) and national fisheries management agencies. The IUCN database combines a variety of data sources compiled by Specialist Groups.
Timing and spatial scale	We selected the ten most recent years (2006 - 2015) of data from the fisheries catch and trade databases. We included all countries listed in the database (spatial scale is global).
Data exclusions	We corrected one species (Coregonus lavaretus) in the supplementary analysis (the secondary global catch database described in Watson & Tidd 2018). This species is likely misidentified because C. lavaretus is only found in freshwater environments, but is commonly confused with other species in the Coregonus genus (https://www.fishbase.se/summary/232). We reassigned this species to "Coregonus." This species is not listed in the primary catch database from the Sea Around Us.<br>We did not show all results in every figure or table: One species (Gadus morhua) was excluded from the final analysis, as explained in the text, but included in Supplementary Table 1 (list of threatened species). In Supplementary Tables 3 and 4, only results for the top 50 countries (by volume of catch and imports, respectively) are shown.
Reproducibility	We tested the final code three times over the course of two weeks to ensure reproducibility, and were successful each time. Additionally, we tested the code in the publicly available GitHub repository to ensure that the figures and results reported in the text can be reproduced.
Randomization	Randomization was not relevant to our study as no experiments were conducted in this study. The study groups in this analysis are predetermined (species and countries); thus, the authors did not allocate the samples (catch and trade records) into groups.
Blinding	Blinding was not inherently relevant to our study. No experiments (including those involving live subjects) were conducted in this study and no treatments were applied. However, the theory of blinding was integrated into the data analysis because the research questions were decided and the entire analysis was outlined before running any code.

Did the study involve field work? ☐ Yes ☒ No

Reporting for specific materials, systems and methods

We require information from authors about some types of materials, experimental systems and methods used in many studies. Here, indicate whether each material, system or method listed is relevant to your study. If you are not sure if a list item applies to your research, read the appropriate section before selecting a response.

Materials & experimental systems

n/a	Involved in the study
[x]	[ ] Antibodies
[x]	[ ] Eukaryotic cell lines
[x]	[ ] Palaeontology
[x]	[ ] Animals and other organisms
[x]	[ ] Human research participants
[x]	[ ] Clinical data

Methods

n/a	Involved in the study
[x]	[ ] ChIP-seq
[x]	[ ] Flow cytometry
[x]	[ ] MRI-based neuroimaging
