# Supplementary material for: Over 90 endangered fish and invertebrates are caught in industrial fisheries
Source: Nat Commun. 2020 Sep 21;11:4764. doi: 10.1038/s41467-020-18505-6 (PMC7506527; doi:10.1038/s41467-020-18505-6)
Supplement: Supplementary file 4 — Description of Additional Supplementary Files [file 41467_2020_18505_MOESM4_ESM.pdf]

Description of Additional Supplementary Files

File Name: Supplementary Movie 1
Description: Short video (3:25) describing the study and key results to the public or a general audience
